# Detection of Autosomal Dominant Polycystic Kidney Disease by Medical Checkup at an Early Stage

**DOI:** 10.7759/cureus.18595

**Published:** 2021-10-08

**Authors:** Shohei Fukunaga, Fumika Kamei, Hirotaka Sonoda, Masafumi Oba, Miharu Kawanishi, Masahiro Egawa, Takafumi Ito, Kazuaki Tanabe

**Affiliations:** 1 Internal Medicine IV, Shimane University Faculty of Medicine, Izumo, JPN

**Keywords:** renal prognosis, ckd, tolvaptan, medical checkup, adpkd

## Abstract

Introduction: Autosomal dominant polycystic kidney disease (ADPKD) is the most common inherited renal disease. Although abdominal echography during medical checkup may be effective for the early detection of ADPKD, there are no reports of the early detection of ADPKD during medical checkup. We investigated whether there was a difference in renal function and total kidney volume (TKV) at the time of diagnosis due to differences in diagnostic triggers for ADPKD.

Methods: A total of 34 patients diagnosed with ADPKD between January 1, 2010, and December 31, 2020, at the Department of Nephrology, Shimane University Hospital, were included. The triggers for diagnosis of the renal cyst(s) were usually unintentional findings. These included findings observed upon routine medical checkups, computed tomography, or abdominal echography during examination for other diseases (incidental detection group) and cases referred to our department for renal dysfunction (renal dysfunction group), and “other” group. We compared the renal dysfunction group and the incidental detection group.

Results: The estimated glomerular filtration rate (eGFR) at diagnosis was significantly higher in the incidental detection group. The TKV was significantly lower in the incidental detection group than in the other group. The number of patients with eGFR > 45 mL/min/1.73 m^2^, for which tolvaptan was safe and effective, was significantly higher in the incidental detection group than in the renal dysfunction group.

Conclusion: Our study shows that medical checkup enables early detection of ADPKD. This is important because ADPKD may have serious complications. The present study did not examine the age at which abdominal echography screening for the early detection of ADPKD was more useful or cost-effective; thus, further research is needed to ascertain this.

## Introduction

Autosomal dominant polycystic kidney disease (ADPKD) is the most common inherited renal disease, with an estimated prevalence of approximately 1 in 2000-4000 [1-3]. Approximately half of the patients with ADPKD develop end-stage renal disease by 60 years of age; therefore, the renal prognosis is poor [4]. Tolvaptan is the only treatment currently available, but its efficacy and safety in patients with an estimated glomerular filtration rate (eGFR) of <45 mL/min/1.73 m^2^ has not been established. Furthermore, if tolvaptan is contraindicated, conservative treatment is preferred; however, early intervention is desirable when renal function still remains preserved.

Medical checkup may be useful for the early detection of ADPKD; however, there are no reports of abdominal echography during medical checkups to detect ADPKD at an early stage. Therefore, we investigated whether there was a difference in renal function and total kidney volume (TKV) at diagnosis due to differences in diagnostic triggers for ADPKD.

## Materials and methods

This retrospective study included the records of patients who were diagnosed with ADPKD during the period of January 1, 2010, to December 31, 2020, at Shimane University Hospital, Japan, to extract information about sex, age, family history of ADPKD, the presence of comorbidities, serum creatinine levels at diagnosis, eGFR, a decline in eGFR, TKV at diagnosis, height-adjusted TKV (HtTKV), rate of increase in TKV, use of tolvaptan, and the introduction of renal replacement therapy.

Kidney volume was measured by computed tomography and calculated using the modified ellipsoid formula: Volume = π/6 × Length × Width × Depth [5]. TKV was obtained by summing the volumes of both kidneys. The decline in eGFR was calculated by dividing the difference between pre-diagnosis and at-diagnosis eGFR values by the duration of the follow-up period in years. The rate of renal volume expansion was calculated using the difference between the TKV at diagnosis and the TKV at follow-up divided by the duration of the follow-up period in years. The cases were categorized as follows: patients diagnosed by medical checkup, computed tomography, or abdominal echography during examination for other diseases (incidental detection group); patients referred to our department because of renal dysfunction, in addition to patients diagnosed with ADPKD (renal dysfunction group) and “other” group.

Comparisons regarding different triggers for ADPKD diagnosis were made between the renal dysfunction group and the incidental detection group. Patients who were lost to follow-up were excluded from the analysis. Furthermore, the rate of increase in TKV, the decrease in eGFR, and the presence or absence of renal replacement therapy were compared between the tolvaptan-treated and non-treated groups to examine the effect of tolvaptan, depending on the diagnostic trigger for ADPKD.

Fisher’s exact test or Student’s t-test was used to analyze the data, and statistical significance was set at p < 0.05. Statistical analyses were performed using Prism 7 version 7.0 (GraphPad Software Inc., San Diego, CA).

This study was performed in accordance with the principles of the Declaration of Helsinki and was approved by the Institutional Ethics Committee of Shimane University Faculty of Medicine (study number 20210324-1). This study is retrospective, and the analysis used anonymous clinical data; therefore, the requirement for informed consent was waived.

## Results

Thirty-four patients (18 male and 16 female patients; mean age, 50.0 ± 16.1 years) were included in this study (Table 1). All the enrolled patients met the criteria of the clinical practice guidelines for the diagnosis and management of ADPKD [6]. Family history of ADPKD was found in 19 patients (29.4%). Complications included eight cases of cerebral aneurysm, one case of subarachnoid hemorrhage, and one case of mitral regurgitation. Seven patients (20.6%) had subjective symptoms at the time of their initial medical consultation. These included five cases of back pain, one case of abdominal discomfort, and one case of perception of an abdominal mass. The eGFR at diagnosis was 63 ± 30.8 mL/min/1.73 m^2^, and the HtTKV was 634.6 ± 547.2 mL/m. Tolvaptan was administered to 12 patients (35.3%) thereafter.

**Table 1:** Characteristics of patients included in the study M: male, F: female, ADPKD: autosomal dominant polycystic kidney disease, eGFR: estimated glomerular filtration rate, TKV: total kidney volume, HtTKV: height-adjusted total kidney volume

Patient characteristics	
Sex (n)	
M	18
F	16
Age (years)	50.0 ± 16.1
Family history of ADPKD (n)	19 (55.9%)
Comorbidities (n)	10 (29.4%)
Subjective symptoms (n)	7 (20.6%)
eGFR at diagnosis (mL/min/1.73 m^2^)	63.0 ± 30.8
TKV at diagnosis (mL)	1041.3 ± 547.2
HtTKV at diagnosis (mL/m)	634.6 ± 547.2
Use of tolvaptan (n)	12 (35.3%)

**Figure 1:**
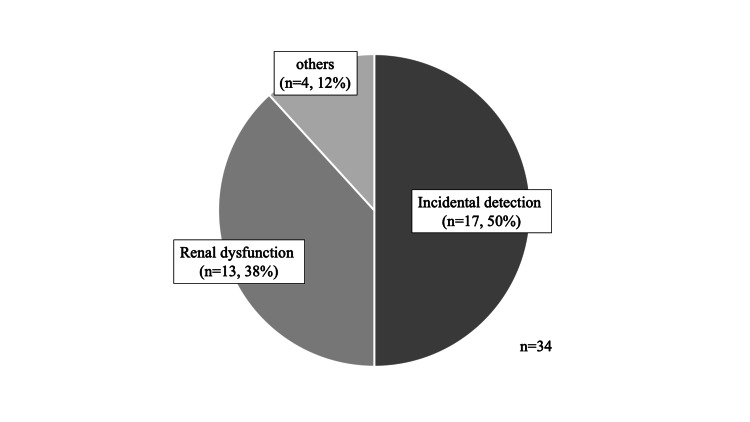
Triggers for autosomal dominant polycystic kidney disease diagnosis

The most common diagnostic trigger for ADPKD was incidental detection (17 patients; 50%), followed by renal dysfunction (13 patients; 38%), and other (4 patients; 12%) (Figure 1). The differences in diagnostic triggers were compared between the renal dysfunction group (n=10) and the incidental detection group (n=16) (Table 2). Patients who were lost to follow-up were excluded from the analysis. There were no significant differences in baseline characteristics including sex, age, family history, comorbidities, use of tolvaptan, and the duration of follow-up between the renal dysfunction and the incidental detection groups. At diagnosis, the eGFR was significantly lower, and the TKV was significantly higher in the renal dysfunction group. The eGFR at diagnosis was ≥45 mL/min/1.73 m^2^ in three patients in the renal dysfunction group and in 16 patients in the incidental detection group. Three patients in the renal dysfunction group developed end-stage renal failure versus none in the incidental detection group. Thus, the renal dysfunction group had a significantly higher end-stage renal failure incidence than the incidental detection group (p = 0.0462).

**Table 2:** Comparison of different triggers for ADPKD diagnosis M: male, F: female, ADPKD: autosomal dominant polycystic kidney disease, eGFR: estimated glomerular filtration rate, TKV: total kidney volume, HtTKV: height-adjusted total kidney volume, RRT: renal replacement therapy Comorbidities included eight cases of cerebral aneurysm, one case of subarachnoid hemorrhage, and one case of mitral regurgitation. *p<0.05, **p<0.0005, ***p<0.0001

	Triggers for ADPKD diagnosis		
	Renal dysfunction	Incidental detection	p
Cases, sex (n)			>0.9999
M	5	8	
F	5	8	
Age (years)	54.4 ± 2.6	46.3 ± 4.7	0.2109
Family history of ADPKD	8 (80.0%)	8 (50.0%)	0.2177
Comorbidities (n)	3 (30.0%)	5 (31.3%)	>0.9999
eGFR at diagnosis (mL/min/1.73 m^2^)	39.7 ± 3.5	85.0 ± 6.8	<0.0001***
eGFR at diagnosis >45 mL/min/1.73 m^2^	3 (30.0%)	16 (100%)	0.0002**
TKV at diagnosis (mL)	1532.1 ±361.8	675.7 ±83.8	0.0091*
HtTKV at diagnosis (mL/m)	905.9 ± 201.3	416.8 ±52.6	0.0103*
Use of tolvaptan (n)	5 (50.0%)	4 (25.0%)	0.2341
Initiation of RRT (n)	3 (30.0%)	0 (0%)	0.0462*
Follow-up period (year)	5.0 ± 0.6	3.4 ± 0.7	0.1061

**Figure 2:**
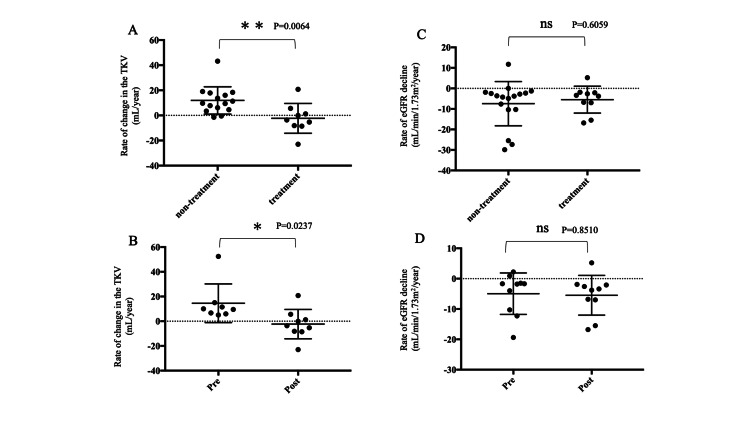
Effect of tolvaptan on the rate of change in TKV and the decline in eGFR eGFR: estimated glomerular filtration rate, TKV: total kidney volume, ns: not significant The rate of change in TKV was compared between the tolvaptan-treated and non-treated groups (A). The rate of change in TKV was compared before and after treatment with tolvaptan (B). The decline in eGFR was compared between the tolvaptan-treated and non-treated groups (C). The decline in eGFR was compared before and after treatment with tolvaptan. *p < 0.05, **p < 0.01. Error bars in bar plots represent standard errors of the mean.

Tolvaptan was initiated in 12 patients (35.2%). Patients who were lost to follow-up were excluded from the analysis. There were no significant differences in baseline characteristics including sex, age, comorbidities, eGFR, and TKV, or follow-up time from the initial diagnosis between the tolvaptan-treated and non-treated groups (Table 3). The duration between ADPKD diagnosis and initiation of tolvaptan was 2.7 ± 2.0 years. The rate of change in TKV was significantly reduced in the tolvaptan-treated group (-2.3 ± 3.9%/year versus +14 ± 5.5%/year before treatment) compared with the non-treated group (+11.9 ± 2.8%/year) (Figure 2, panels A, B).

**Table 3:** Comparison between patients treated with and without tolvaptan M: male, F: female, ADPKD: autosomal dominant polycystic kidney disease, eGFR: estimated glomerular filtration rate, TKV: total kidney volume, HtTKV: height-adjusted total kidney volume, RRT: renal replacement therapy

	Use of tolvaptan		
	Yes	No	p
Cases, sex (n)			0.1201
M	7	6	
F	3	11	
Age (years)	44.6 ± 3.3	51.4 ± 4.4	0.2918
Family history of ADPKD	6 (60.0%)	11 (64.7%)	>0.9999
Comorbidities	2 (20.0%)	6 (35.3%)	0.6655
eGFR at diagnosis (mL/min/1.73m^2^)	62.5 ± 7.5	72.2 ± 8.5	0.4478
TKV at diagnosis (mL)	1053 ±164.8	951.6 ± 239.8	0.7666
HtTKV at diagnosis (mL/m)	620.9 ± 94.3	591.1 ± 143.2	0.8811
Duration of the diagnosis and tolvaptan start (year)	2.7 ± 2.0	-	-
Follow-up period from the time of diagnosis (year)	4.5 ± 0.8	3.7 ± 0.6	0.4489

The decline in eGFR was -5.5 ± 2.1 mL/min/1.73 m^2^/year in the tolvaptan-treated group and -7.4 ± 2.6 mL/min/1.73 m^2^/year in the non-treated group (Figure 2, panel C). This change was not significant as the decline in eGFR before treatment was -5.5 ± 2.1 mL/min/1.73 m^2^/year (Figure 2, panel D). There was a significant difference between the renal dysfunction group (39.7 ± 3.5 mL/min/1.73 m^2^) and the incidental detection group (85.0 ± 6.8 mL/min/1.73 m^2^) (p < 0.0001).

The mean duration of treatment with tolvaptan was 1.8 ± 0.9 years, and the mean dose was 80.3 ± 22.8 mg/day. Among the patients treated with tolvaptan, five were in the renal dysfunction group, four were in the incidental detection group, and one was in the other group. TKV augmentation before and after treatment with tolvaptan was -5.0 ± 5.0%/year in the renal dysfunction group and 2.5 ± 9.2%/year in the incidental detection groups, which was not statistically significant (p = 0.4653). The decline in eGFR was measured in five patients in the renal dysfunction group, four patients in the incidental detection groups, and one patient in the other group. The decline in eGFR was -2.2 ± 2.0 mL/min/1.73 m^2^/year in the renal dysfunction group and -9.3 ± 4.0 mL/min/1.73 m^2^/year in the incidental detection groups, which was not statistically significant (p = 0.1355).

## Discussion

Compared to the incidental detection group, the renal dysfunction group had a poorer renal function and a higher TKV. Age was also less in the incidental detection group compared to the renal dysfunction group, considering that ADPKD progresses with age. These findings suggest that the early detection of ADPKD via medical checkup is important prior to the decline in renal function.

Currently, tolvaptan is the only drug available for the treatment of ADPKD. Tolvaptan has been proven to inhibit the decline in eGFR by 0.92 mL/min/1.73 m^2^/year and the rate of increase in TKV in the Tolvaptan Efficacy and Safety in Management of Autosomal Dominant Polycystic Kidney Disease and Its Outcomes (TEMPO) 3:4 study [7]. Tolvaptan is indicated only for patients with a TKV ≥750 mL and a kidney volume augmentation rate of approximately 5%/year or more. Furthermore, the safety and efficacy of tolvaptan have not been established in ADPKD patients with an eGFR <45 mL/min/1.73 m^2^, and it is contraindicated in patients with an eGFR <15 mL/min/1.73 m^2^. There were no patients with an eGFR <15 mL/min/1.73 m^2^ in this study. However, only 38.5% of the patients in the renal dysfunction group had an eGFR >45 mL/min/1.73 m^2^, for which the safety and efficacy of tolvaptan have been established. In contrast, as many as 94.1% of the patients in the incidental detection groups had an eGFR >45 mL/min/1.73 m^2^, for which tolvaptan could be safely used.

In this study, there was no difference in the effect of tolvaptan according to the trigger for ADPKD diagnosis. This could be due to the short follow-up period (mean 1.8 ± 0.9 years) and the lower dose of tolvaptan, which was 80 mg/day in this study versus 95 mg/day in the TEMPO 3:4 study. Regardless, the rate of increase in TKV was significantly suppressed, suggesting that the effects of continued tolvaptan will become apparent in the future. In addition, the TEMPO 4:4 study showed that the pharmacological effects of tolvaptan are sustained and cumulative in patients receiving long-term treatment [8].

Early detection of ADPKD and intervention by a nephrologist is recommended, even when tolvaptan is not administered. Although there are no reports showing that early detection and intervention by nephrologists improves renal prognosis in patients with ADPKD, this approach has been shown to improve the prognosis in patients with chronic kidney disease (CKD) [9,10]. In addition, it has been reported that educating patients with CKD is important, and dietary guidance for CKD patients is cost-effective [11,12].

In this study, among the 26 patients who were followed up, the likelihood of end-stage renal failure was significantly higher in the renal dysfunction group, suggesting that the early detection of ADPKD may improve renal prognosis. This was a retrospective study and there is no long-term comparison of renal prognosis by early detection via medical checkup; therefore, further research is needed.

Renal cysts are a relatively common finding on abdominal echography during medical checkups; therefore, it is difficult to closely examine them all. On abdominal echography at medical checkup, it has been reported that about half of the patients with more than five cysts in each kidney had ADPKD [13]. The Japanese Manual for Abdominal Ultrasound in Cancer Screening and Health Checkups also states that the presence of five or more cysts in both kidneys should be considered as requiring further thorough examination [14].

## Conclusions

In conclusion, medical checkup is associated with early detection of ADPKD patients with small total kidney volume and better renal function. Early detection of ADPKD is important because ADPKD may have serious complications. The present study did not examine the age at which abdominal echography screening for the early detection of ADPKD was more useful or cost-effective; thus, further research is needed to ascertain these. We hope that early detection and intervention through proactive screening tests will be possible in the future.
